# Prognostic factors and clinical efficacy of second-line treatments of *Pneumocystis jirovecii* pneumonia for non-HIV patients after first-line treatment failure

**DOI:** 10.1186/s12879-022-07523-y

**Published:** 2022-06-14

**Authors:** Anlei Liu, Ruixue Sun, Guanghui Cao, Xiaohang Liu, Huadong Zhu, Jing Yang

**Affiliations:** 1 Emergency Department, State Key Laboratory of Complex Severe and Rare Diseases, Peking Union Medical College Hospital, Chinese Academy of Medical Science and Peking Union Medical College, Beijing, 100730 China; 2 Psychological Department, State Key Laboratory of Complex Severe and Rare Diseases, Peking Union Medical College Hospital, Chinese Academy of Medical Science and Peking Union Medical College, Beijing, 100730 China; 3 Cardiology Department, State Key Laboratory of Complex Severe and Rare Diseases, Peking Union Medical College Hospital, Chinese Academy of Medical Science and Peking Union Medical College, Beijing, 100730 China

**Keywords:** *Pneumocystis jirovecii* pneumonia, Non-HIV immunocompromised patients, Drug resistance

## Abstract

**Background:**

*Pneumocystis jirovecii* pneumonia (PCP) is a life-threatening opportunistic infection. In non-HIV immunocompromised patients with PCP, a standard second-line treatment has not been established up to now.

**Methods:**

Non-HIV immunocompromised patients with confirmed PCP between April 2013 and December 2020 were included. Their PCP treatment history was tracked. Factors related to first-line trimethoprim/sulfamethoxazole (TMP/SMX) and second-line treatment failure were identified. Different second-line treatment strategies were compared.

**Results:**

Among the 220 patients, 127 (57.73%) did not respond to first-line TMP/SMX treatment. Risk factors related to treatment failure included symptom triad with breathlessness at rest, persistent fever and cough (85% in the treatment failure group versus 74% in the treatment success group, *P* = 0.034), treatment with invasive mechanical ventilation (67 vs. 19%, *P* < 0.001), coinfection with CMV (69 vs. 47%, *P* = 0.035), and bacteremia (59 vs. 10%, *P* < 0.001). A total of 49 patients received second-line treatment on the basis of TMP/SMX, and 28 (57.1%) of them responded to the treatment. No clinical parameter, including selection of different therapies, was found to be significantly associated with second-line treatment failure. Further, the prognosis of different second-line therapies showed no drug or drug combination strategy superior to others. The primaquine group had lower 90-day mortality rate (45.9%) but showed no statistically significant difference compared with the non-primaquine group (64.6%). The patients in the clindamycin plus primaquine group had the lowest in-hospital mortality rate (22.2%, *P* = 0.042) among different second-line therapies, although the in-hospital mortality of the primaquine group was not significantly different from that of the non-primaquine group. The differences in 28 day mortality and overall mortality rates were not statistically significant, too.

**Conclusion:**

CMV infection and bacteremia were risk factors significantly associated with treatment failure of TMP/SMX. The response and survival rates of second-line treatment, including clindamycin, primaquine, and caspofungin, were poor, maybe clindamycin plus primaquine as second line treatment was better than other treatment strategies. These results suggest that clinicians should carefully evaluate whether the treatment of TMP/SMX has failed due to a coinfection rather than hastily changing to a second-line drug when the patient worsens.

## Introduction

*Pneumocystis jirovecii* pneumonia (PCP) is a life-threatening opportunistic infection that often occurs in immunocompromised patients [1]. On one hand, although PCP most commonly occurs in HIV-positive patients, with the increased use of new HIV antiretroviral medications and routine prophylaxis of PCP, its incidence has decreased gradually [2]. On the other hand, the expanding use of immunosuppressive medications for patients with solid and hematological malignancies, the development of organ or hematopoietic stem cell transplants, and the increased prevalence of autoimmune and inflammatory diseases has made PCP a prevalent opportunistic infection among patients without HIV infection [3]. Furthermore, non-HIV immunocompromised patients are characterized by a more rapid disease progression, with a mortality rate of 35–55% compared with 10–20% in HIV-infected patients [3, 4].

Based on data from clinical trials, trimethoprim/sulfamethoxazole (TMP/SMX) is the first-line medications for PCP-infected patients because of its low cost and good clinical efficacy, but when clinicians are faced with its clinical failure, they have to switch drug therapy to second-line agents. The alternative treatment regimens included clindamycin with primaquine, dapsone with trimethoprim, atovaquone, or pentamidine [4, 5]. Recently, caspofungin was also found capable of slowing down or even stopping PCP progression via substantial depletion of the fungal burden [6]. However, there is no widely accepted consensus on an optimal second-line regimen. Meanwhile, the majority of information on PCP treatment is extrapolated from HIV-related studies, with limited literature that has determined the efficacy in the non-HIV population [6, 7].

The goal of this study was to explore the risk factors for treatment failure during TMP/SMX therapy for PCP and to compare the efficacy of the available second-line agents, including clindamycin, primaquine and caspofungin.

## Methods

### Patients

We conducted a retrospective study at Peking Union Medical College Hospital (PUMCH). The study cohort comprised all non-HIV immunocompromised patients who had confirmed PCP and were treated at PUMCH between April 1st, 2013, and December 31st, 2020.

### Data collection

From the patients’ medical records, we collected their information, including age, sex, underlying diseases (autoimmune disease, hematological malignancies, solid tumor, organ transplantation and others), treatment history (glucocorticoid, immunosuppressant and preventive dose of sulfanilamide, PCP prophylaxis), clinical manifestations (fever, cough, wheeze), level of white blood cells (WBC), lymphocyte (LY#), lactate dehydrogenase (LDH) and (1–3)-beta-D-glucan (BDG), need for ICU and mechanical ventilation, dates of treatment initiation and cessation, treatment switches, complications [bacteremia, cytomegalovirus (CMV) infection] and outcome.

### PCP treatment

During this 8-year period, primary PCP episodes in non-HIV immunocompromised patients were treated with TMP/SMX initiated at a standard dosage of 15–20 mg/kg/day of TMP equivalent in 3–4 doses per day. The duration of TMP/SMX treatment was 21 days for patients who responded. If the patient was previously allergic to sulfonamides, desensitization therapy would be adopted. If acute kidney injury appeared, the dose of TMP/SMX was adjusted according to the renal function.

**Fig. 1:**
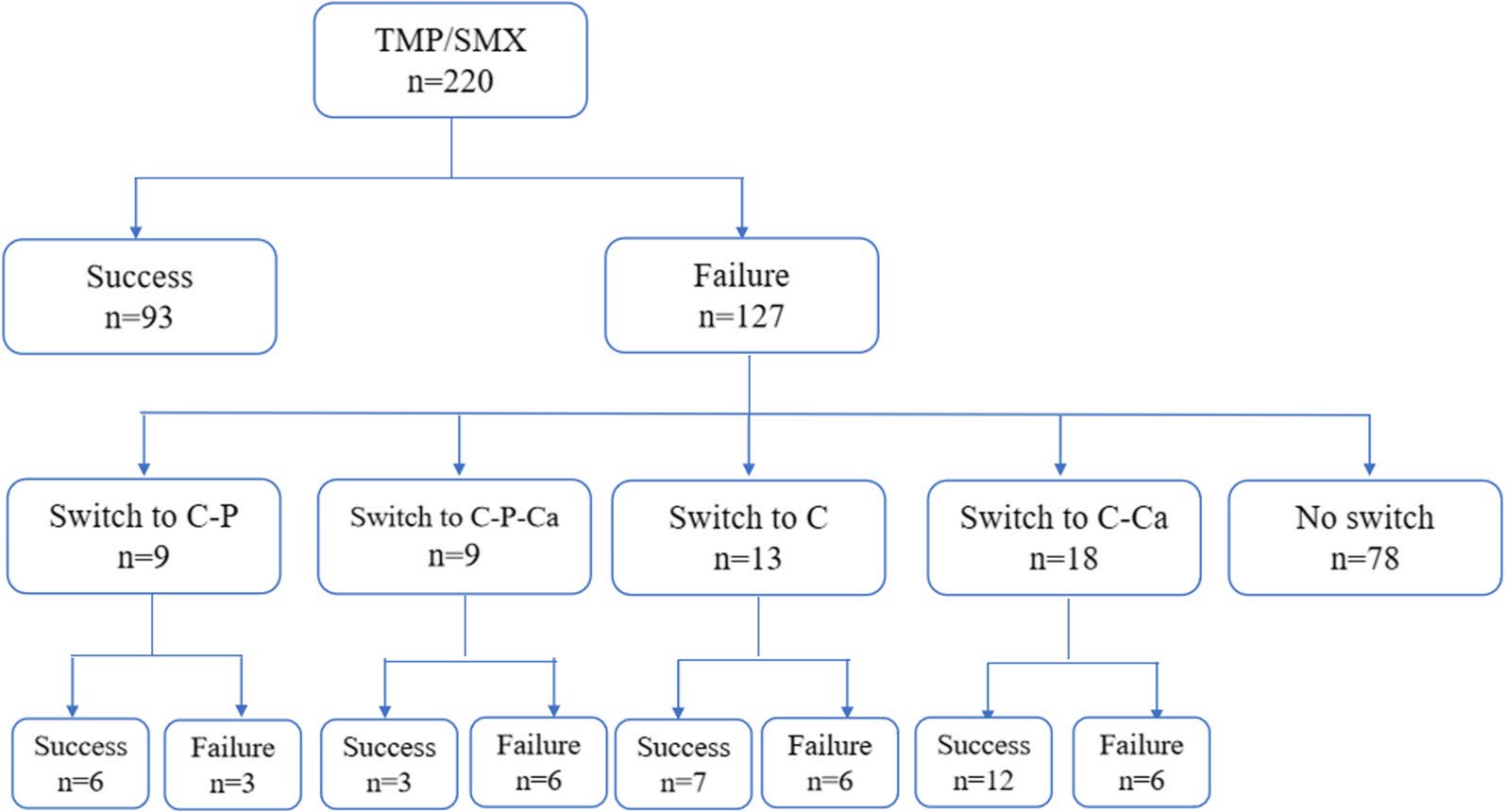
Patients’ treatment flow and outcome. Clindamycin (C), clindamycin plus primaquine (C-P), clindamycin plus caspofungin (C-Ca), clindamycin plus primaquine plus caspofungin (C-P-Ca)

In cases of treatment failure, a second-line regimen could be used according to the clinician’s consideration. Because of the unavailability of most regimens and the lack of clinical experience, the second-line treatments used are shown in Fig. 1, including clindamycin (1200 mg, intravenous infusion, 2 times daily) (C), clindamycin (600 mg, oral, 3 times daily) plus primaquine (30 mg, oral, once daily) (C-P), clindamycin (1200 mg, intravenous infusion, 2 times daily) plus caspofungin (50 mg, intravenous infusion, once daily) (C-Ca), and clindamycin (1200mg, intravenous infusion, 2 times daily) plus primaquine (30 mg, oral, once daily) plus caspofungin (50 mg, intravenous infusion, once daily) (C-P-Ca).

For patients with severe infection, adjunctive corticosteroids (40–80 mg daily) were given until PCP improvement. Except for steroids, other disease-related immunosuppressive drugs were stopped.

### Definition

The eligible patients had clinical manifestations of PCP, such as dyspnea, cough, fever, or abnormal chest radiographs. The mycological and morphological diagnoses were confirmed by real-time fluorescence quantitative polymerase chain reaction (qPCR) and Grocott's methenamine silver (GMS) staining of respiratory samples, respectively [8]. Based on the criteria used in previous studies [5, 9, 10], the diagnosis of PCP was considered definitive if a positive PCR test for *Pneumocystis jirovecii* (*P. jirovecii*) DNA in bronchoalveolar lavage fluid (BALF) or GMS staining positive on BALF or sputum or a positive PCR test for *P. jirovecii* DNA in sputum was confirmed, combined with an increased level of BDG from patients with clinical manifestations (fever, dry cough, or dyspnea), hypoxemia, and radiologic findings compatible with PCP. The diagnosis of PCP was considered presumptive if patients met all clinical criteria and had either a positive PCR test for *P. jirovecii* DNA in sputum or an increased level of BDG, after excluding other fungal infections [9]. In our study, both definitive and presumptive cases were included.

Treatment success was defined as a clinical cure or showing definitive clinical improvement with resolution of dyspnea and chest infiltrates. Treatment failure was defined as clinical deterioration occurring 4–8 days after TMP/SMX treatment: (1) progressive clinical deterioration characterized by an inability to maintain a stable PaO_2_ despite an increased proportion of FiO_2_ and/or persistent fever; and (2) progressive deterioration of vital signs and/or radiographic worsening [11]. The classification of the PCP severity is based on clinical symptoms, arterial blood gases and oxygen saturation, and radiological findings [12]. CMV infection was defined as having CMV blood viral load of over 500 copies/ml, and all such patients received ganciclovir treatment.

The primary outcome was 90-day mortality, and the secondary outcomes included in-hospital mortality, 28-day mortality and overall survival, defined as the time from diagnosis to death or last follow-up.

### Statistics

Risk factors associated with TMP/SMX treatment failure were analyzed by Mann–Whitney U or Fisher’s exact tests as appropriate. To identify independent risk factors, parameters with P < 0.05 in the univariate analysis were analyzed using a multivariate logistic regression model. Categorical variables were compared using the chi-square test. Kaplan–Meier analysis was used for survival analysis, and survival curves of different groups were compared using the log-rank test. P < 0.05 was considered statistically significant. Considering the limited sample size of our study, the standardized mean difference (SMD) was used to describe the effect after controlling the baseline confounding factors and to reflect the differences between groups if the *p*-value was > 0.05. SPSS version 22.0 (IBM Corporation, Armonk, New York, USA) was used for the statistical analysis.

## Results

### Identifying risk factors associated with first-line TMP/SMX treatment failure

Between April 1st, 2013, and December 31st, 2020, 220 non-HIV immunocompromised patients infected with PCP were hospitalized in our hospital. Among these 220 patients, 182 had definite PCP infection, and 38 had presumptive PCP infection. All of the patients were first treated with TMP/SMX. Acute kidney injury occurred in 35 of the 220 patients, and 8 of these patients were previously allergic to sulfonamides. Despite the adverse reactions, they still managed to use TMP/SMX after dose adjustment and desensitization therapy. Among these 220 patients, 127 (57.73%) did not respond to the first-line TMP/SMX treatment.

Complete clinical data were available in 209 patients. We compared the clinical characteristics between the treatment success group (n = 93) and the treatment failure group (n = 116) as shown in Table 1. The risk factors related to TMP/SMX treatment failure in PCP-infected non-HIV immunocompromised patients included symptom triad with breathlessness at rest, persistent fever and cough (*P* = 0.034), treatment with invasive mechanical ventilation (*P* < 0.001), coinfection with CMV (*P* = 0.035), and bacteremia (*P* < 0.001).

**Table 1:** Risk factors for TMP/SMX treatment failure in PCP infected non-HIV immunocompromised patients by univariate analysis and multivariate analysis

	Univariate analysis			Multivariate analysis		
Parameters	Failure, n = 116	Success, n = 93	*P* value	*P* value	OR	95% CI
Demographic information						
Male, n (%)	55 (47)	52 (56)	0.222			
Age, yr (range)	55 (44–65)	50 (31–62)	0.011			
Underlying disease						
RID, n (%)	77 (61)	46 (44)	0.009			
Hematologic tumor, n (%)	8 (6.4)	6 (5.7)	0.840			
Solid tumor, n (%)	6 (4.8)	4 (3.8)	0.976			
Organ transplant, n (%)	6 (4.8)	1 (1)	0.199			
Other disease, n (%)	32 (25)	47 (45)	0.002			
TS, n (%)	107 (85)	78 (74)	0.044	0.034	0.356	0.138–0.923
Lab examination						
WBC, × 10.^9^/L [IQR]	8.2 [6.4]	8 [5.5]	0.814			
LY, × 10.^9^/L [IQR]	0.5 [0.6]	0.8 [0.8]	0.001			
LDH, U/L [IQR]	577 [330]	447 [275]	< 0.001			
BDG, pg/ml [IQR]	571 [1510]	750 [1006]	0.362			
Treatment						
Sufficient steroid, n (%)	70 (56)	57 (54)	0.847			
Steroid course > 3 m, n (%)	81 (64)	68 (65)	0.940			
Immunosuppressant, n (%)	97 (77)	85 (81)	0.463			
Preventive TMP/SMX, n (%)	27 (21)	21 (20)	0.790			
IMV, n (%)	84 (67)	20 (19)	< 0.001	< 0.001	0.222	0.103–0.48
Co-infection						
CMV, n (%)	87 (69)	49 (47)	0.001	0.035	0.464	0.227–0.947
Bacteremia, n (%)	74 (59)	10 (10)	< 0.001	< 0.001	0.148	0.060–0.367

*TMP/SMX* trimethoprim/sulfamethoxazole, *PCP* *Pneumocystis jirovecii* pneumonia, *HIV* human immunodeficiency virus, *OR* odds ratio, *CI* confidence interval; *RID* rheumatic immune disease, *TS* typical symptoms of simultaneous breathlessness at rest, persistent fever and cough; *SD* standard deviation, *WBC* white blood cell; *IQR* interquartile range, *LY* lymphocyte, *LDH* lactate dehydrogenase; *BDG* (1–3)-beta-D-glucan, *IMV* Invasive mechanical ventilation, *CMV* Cytomegalovirus

### Risk factors associated with second-line treatment failure

Among the patients who did not respond to TMP-SMX, 78 died after not being given an alternative regimen, and 71 died during the administration of TMP/SMX. In the remaining 49 patients, 42 had definite PCP infection, and 7 had presumptive PCP infection. They received second-line treatment: C-P for 9 patients, C-P-Ca for 9 patients, C for 13 patients and C-Ca for 18 patients. In all, 57.1% (28/49) patients responded to the treatment, 42.9% (21/49) patients did not respond to the second-line therapies. According to different therapies, the response rates were 66.67% (6/9) in the C-P group, 33.3% (3/9) in the C-P-Ca group, 53.85% (7/13) in the C group, and 66.67% (12/18) in the C-Ca group. As shown in Table 2, the comparison of clinical features between second-line treatment success and treatment failure groups revealed no risk factor, including the factors associated with first-line TMP/SMX treatment failure.

**Table 2:** Risk factors for second-line treatment failure in PCP infected non-HIV immunocompromised patients by univariate analysis and multivariate analysis

Parameters	Univariate analysis		
	Failure, n = 21	Success, n = 28	*P* value
Demographic information			
Male, n (%)	12 (57)	10 (36)	0.139
Age, yr (range)	50 (26–74)	48 (18–71)	0.678
Underlying disease			
RID, n (%)	13 (62)	15 (54)	0.560
Tumor, Organ transplant, and other diseases, n (%)	8 (38)	13 (46)	0.560
TS, n (%)	15 (71)	20 (71)	1.000
Lab examination			
WBC, × 10.^9^/L [IQR]	8.1 [5.4]	9.2 [6.3]	0.276
LY, × 10.^9^/L [IQR]	0.5 [0.4]	0.8 [0.8]	0.053
LDH, U/L [IQR]	607 [333]	546 [281]	0.092
BDG, pg/ml [IQR]	924 [1109]	1325 [1749]	0.715
Treatment			
C, n (%)	6 (29)	7 (25)	0.779
C-Ca, n (%)	6 (29)	12 (43)	0.307
C-P, n (%)	3 (14)	6 (21)	0.525
C-P-Ca, n (%)	6 (29)	3 (11)	0.122
Co-infection			
CMV, n (%)	15 (71)	19 (68)	0.788
Bacteremia, n (%)	8 (38)	13 (46)	0.560

*PCP* *Pneumocystis jirovecii* pneumonia, *HIV* human immunodeficiency virus, *OR* odds ratio, *CI* confidence interval, *RID* rheumatic immune disease, *TS* typical symptoms of simultaneous breathlessness at rest, persistent fever and cough; *SD* standard deviation, *WBC* white blood cell, *IQR* interquartile range, *LY* lymphocyte, *LDH* lactate dehydrogenase, *BDG* (1–3)-beta-D-glucan, *IMV* Invasive mechanical ventilation, *CMV* Cytomegalovirus

### Prognosis of different second-line therapies

As the sample sizes of these 4 groups were not large enough to conduct statistical analyses and all of the patients received clindamycin, the 49 patients were divided according to whether primaquine or caspofungin was used: the caspofungin group (n = 27) including patients treated with C-Ca and C-P-Ca, and the noncaspofungin group (n = 22) including patients treated with C and C-P; the primaquine group (n = 18) including patients treated with C-P and C-P-Ca, and the nonprimaquine group (n = 31) including patients treated with C and C-Ca.

**Fig. 2:**
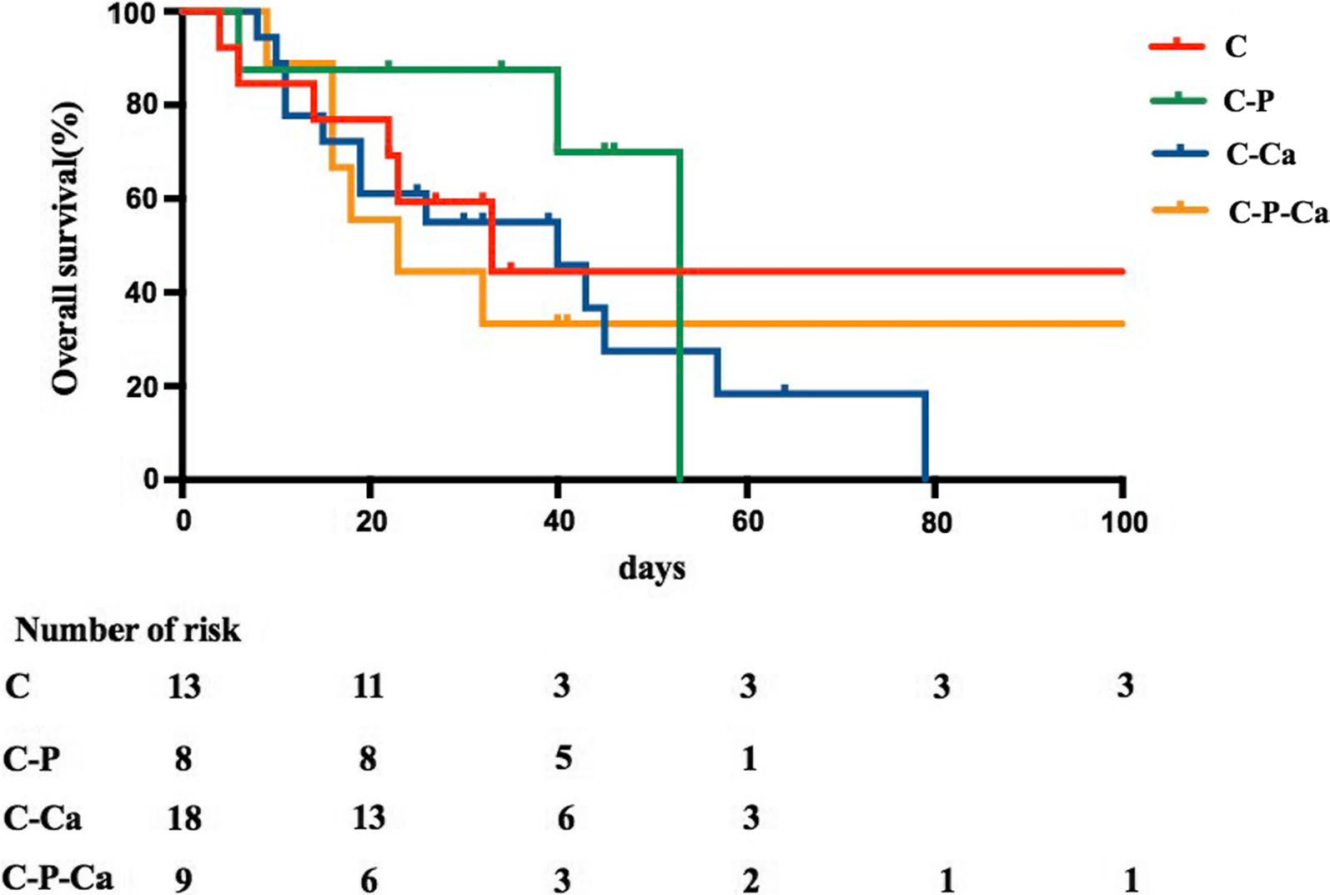
Kaplan–Meier analysis of patients’ overall survival. Clindamycin (C), clindamycin plus primaquine (C-P), clindamycin plus caspofungin (C-Ca), clindamycin plus primaquine plus caspofungin (C-P-Ca)

The characteristics of these groups are summarized in Tables 3 and 4. During the median follow-up time of 44 days, the median overall survival of patients treated with C, C-P, C-Ca, and C-P-Ca was 33 days, 53 days, 40 days, and 23 days, respectively (P = 0.614) (Fig. 2). The 90-day mortality rate of the primaquine group (45.9%) was numerically lower than that of the nonpraquine group (64.6%), but the difference was not statistically significant [*P* = 0.171, SMD = 0.381, odds ratio (OR) = 0.467]. The overall in-hospital mortality rate was 53.1% (26/49), which was the lowest in the C-P group (22.2% in the C-P group, 46.2% in the C group, 66.7% in the C-Ca group, 66.7% in the C-P-Ca group; *P* = 0.042), although the in-hospital mortality of the primaquine group was not significantly different from that of the nonprimaquine group. Similarly, other secondary outcomes including 28-day mortality and overall survival also numerically improved in the primaquine group but the differences were not statistically significant (Table 5). The 90-day mortality was not improved in the caspofungin group, and it was even higher compared with the non-caspofungin group (64.6 vs.21.8%, *P* = 0.003, SMD = 0.958, OR = 6.549). In-hospital mortality, 28-day mortality and overall survival also significantly worsened in the caspofungin group (Table 5).

**Table 3:** The characteristics of primaquine group and non-primaquine group

	Non primaquine (n = 31)	Primaquine (n = 18)	t/χ.^2^ value	*P* value	SMD
	Before weighted				
Male, n (%)	13 (42)	9 (50)	0.299	0.584	0.162
Age, yr ± SD	45.9 ± 18.7	54.3 ± 16.6	1.583	0.120	0.477
RID, n (%)	19 (61)	9 (50)	0.593	0.441	0.229
SS, n (%)	15 (48)	12 (67)	1.538	0.215	0.376
Steroid > 3 m, n (%)	20 (65)	13 (72)	0.308	0.579	0.166
IS, n (%)	25 (81)	15 (83)	0.000	1.000	0.070
p T/S, n (%)	7 (23)	1 (6)	1.331	0.249	0.505
TS, n (%)	21 (68)	14 (78)	0.562	0.453	0.227
WBC, × 10.^9^/L ± SD	9.0 ± 4.2	8.6 ± 4.1	0.375	0.710	0.111
LY, × 10.^9^/L ± SD	0.8 ± 0.6	0.6 ± 0.6	0.848	0.401	0.249
LDH, U/L ± SD	565.5 ± 205.0	637.4 ± 289.7	1.014	0.316	0.286
BDG, pg/ml ± SD	1210.9 ± 1015.3	786.5 ± 1010.7	− 1.413	0.164	0.419
T/S to 2nd days, n (%)	7.2 ± 5.6	5.9 ± 5.1	0.000	1.000	0.233
Severe PCP, n (%)	23 (74)	13 (72)	0.000	1.000	0.045
CMV, n (%)	23 (74)	11 (61)	0.918	0.338	0.282
Bacteremia, n (%)	16 (52)	5 (28)	2.642	0.104	0.502
	After weighted				
Male, n (%)	12.7 (41)	6.8 (38)	0.054	0.816	0.070
Age, yr ± SD	47.8 ± 17.4	51.7 ± 14.8	0.807	0.424	0.243
RID, n (%)	17.1 (55)	9.2 (51)	0.082	0.774	0.084
SS, n (%)	16.6 (53)	8.2 (45)	0.293	0.588	0.162
Steroid > 3 m, n (%)	21.6 (70)	12.4 (69)	0.004	0.951	0.019
IS, n (%)	25.8 (83)	12.8 (71)	1.005	0.316	0.293
p T/S, n (%)	5.3 (17)	4.1 (23)	0.129	0.758	0.141
TS, n (%)	23.0 (74)	14.9 (83)	0.401	0.535	0.200
WBC, × 10.^9^/L ± SD	8.7 ± 4.5	9.5 ± 3.7	0.651	0.518	0.198
LY, × 10.^9^/L ± SD	0.7 ± 0.6	0.6 ± 0.6	− 0.269	0.790	0.078
LDH, U/L ± SD	590.8 ± 200.2	623.5 ± 247.3	0.509	0.613	0.145
BDG, pg/ml ± SD	1066.3 ± 983.3	1056 ± 1204.7	0.036	0.971	0.009
T/S to 2nd days, n (%)	6.7 ± 5.2	5.7 ± 4.4	0.663	0.511	0.200
Severe PCP, n (%)	23.2 (75)	14.3 (79)	0.134	0.731	0.110
CMV infection, n (%)	22.8 (73)	13.5 (75)	0.015	0.903	0.037
Bacteremia, n (%)	13.8 (44)	9.7 (54)	0.428	0.513	0.196

*SMD* standardized mean difference, *RID* rheumatic immune disease, *SS* sufficient steroid, *IS* immunosuppressant, *p T/S* preventive TMP/SMX, *TS* typical symptoms of simultaneous breathlessness at rest, persistent fever and cough, *WBC* white blood cell, *LY* lymphocyte, *LDH* lactate dehydrogenase, *BDG* (1–3)-beta-D-glucan, *T/S to 2nd days* TMP/SMX to second-line therapy days, *PCP* *Pneumocystis jirovecii* pneumonia, *CMV* Cytomegalovirus

**Table 4:** The characteristics of caspofungin group and non-caspofungin group

	Non caspofungin (n = 22)	Caspofungin (n = 27)	t/χ.^2^ value	*P* value	SMD
	Before weighted				
Male, n (%)	11 (50)	11 (41)	0.420	0.517	0.187
Age, yr ± SD	48.7 ± 18.0	49.3 ± 18.8	-0.109	0.914	0.031
RID, n (%)	13 (59)	15 (56)	0.062	0.804	0.072
SS, n (%)	9 (41)	18 (67)	3.251	0.071	0.535
Steroid > 3 m, n (%)	14 (64)	19 (70)	0.250	0.617	0.144
IS, n (%)	17 (77)	23 (85)	0.116	0.733	0.204
p T/S, n (%)	4 (18)	4 (15)	0.000	1.000	0.091
TS, n (%)	17 (77)	18 (67)	0.668	0.414	0.238
WBC, × 10.^9^/L ± SD	9.5 ± 4.0	8.3 ± 4.3	1.054	0.297	0.304
LY, × 10.^9^/L ± SD	0.7 ± 0.5	0.7 ± 0.6	− 0.023	0.981	0.007
LDH, U/L ± SD	639.1 ± 196.7	553.5 ± 266.4	1.252	0.217	0.365
BDG, pg/ml ± SD	1368.3 ± 1123.8	799.6 ± 874.8	1.993	0.052	0.565
T/S to 2nd days, n (%)	5.0 ± 5.1	8.2 ± 5.4	− 2.096	0.041	0.640
Severe PCP, n (%)	16 (73)	20 (74)	0.011	0.915	0.030
CMV, n (%)	16 (73)	18 (67)	0.210	0.647	0.132
Bacteremia, n (%)	6 (27)	15 (56)	3.960	0.047	0.599
	After weighted				
Male, n (%)	6.8 (30.8)	12.4 (45.8)	1.306	0.253	0.313
Age, yr ± SD	50.1 ± 13.0	50.0 ± 19.1	− 0.145	0.979	0.008
RID, n (%)	15.7 (71.3)	14.8 (55.0)	1.312	0.252	0.344
SS, n (%)	14.3 (65.1)	15.3 (56.8)	0.404	0.525	0.172
Steroid > 3 m, n (%)	17.7 (80.7)	19.3 (71.4)	0.534	0.465	0.218
IS, n (%)	19.6 (89.3)	22.2 (82.4)	0.495	0.482	0.198
p T/S, n (%)	1.6 (7.4)	3.0 (11.2)	0.046	0.83	0.132
TS, n (%)	14.9 (67.6)	19.8 (73.4)	0.384	0.561	0.147
WBC, × 10.^9^/L ± SD	11.5 ± 3.8	10.8 ± 4.3	− 0.394	0.772	0.261
LY, × 10.^9^/L ± SD	0.8 ± 0.5	0.7 ± 0.7	0.662	0.643	0.142
LDH, U/L ± SD	539.0 ± 173.4	559.7 ± 305.9	− 0.215	0.822	0.083
BG, pg/ml ± SD	735.6 ± 1020.4	867.9 ± 974.2	0.296	0.735	0.133
T/S to 2nd days, n (%)	12.6 ± 9.9	10.4 ± 5.1	− 0.989	0.304	0.282
Severe PCP, n (%)	13.3 (60.3)	18.0 (66.7)	0.845	0.356	0.249
CMV, n (%)	17.2 (80.0)	17.4 (64.6)	1.188	0.276	0.299
Bacteremia, n (%)	8.6 (38.9)	11.6 (42.8)	0.978	0.356	0.134

*SMD* standardized mean difference, *RID* rheumatic immune disease, *SS* sufficient steroid, *IS* immunosuppressant, *p T/S* preventive TMP/SMX; *TS* typical symptoms of simultaneous breathlessness at rest, persistent fever and cough, *WBC* white blood cell, *LY* lymphocyte, *LDH* lactate dehydrogenase, *BDG* (1–3)-beta-D-glucan; *T/S to 2nd days* TMP/SMX to second-line therapy days, *PCP* *Pneumocystis jirovecii* pneumonia, *CMV* Cytomegalovirus

**Table 5:** 90-day mortality and other secondary outcomes in primaquine versus non-primaquine group, and caspofungin versus non-caspofungin group

	Non-primaquine, n = 31	Primaquine, n = 18	χ.^2^ value	*P* value	SMD	OR
Before controlled						
90-day mortality, n (%)	19 (61.3)	10 (55.6)	0.155	0.926	0.117	0.789 (0.243, 2.563)
In-hospital mortality, n (%)	18 (58.1)	9 (50.0)	0.299	0.803	0.162	0.722 (0.225, 2.321)
28-day mortality, n (%)	13 (41.9)	7 (38.9)	0.044	1.000	0.062	0.881 (0.269, 2.885)
Overall death, n (%)	20 (64.5)	11 (61.1)	0.057	1.000	0.070	0.864 (0.260, 2.869)
After controlled						
90-day mortality, n (%)	20.0 (64.6)	8.3 (45.9)	1.873	0.171	0.381	0.467 (0.143, 1.517)
In-hospital mortality, n (%)	19.0 (61.1)	7.9 (43.7)	1.306	0.253	0.355	0.493 (0.153, 1.596)
28-day mortality, n (%)	14.3 (46.0)	6.3 (34.8)	0.660	0.417	0.229	0.627 (0.190, 2.070)
Overall death, n (%)	21.1 (67.9)	8.8 (48.8)	1.510	0.219	0.394	0.451 (0.137, 1.481)

	Non caspofungin, n = 22	Caspofungin, n = 27	χ.^2^ value	*P* value	SMD	OR
Before controlled						
90-day mortality, n (%)	10 (45.5)	19 (70.4)	3.115	0.078	0.522	2.850 (0.878, 9.252)
In-hospital mortality, n (%)	9 (40.9)	18 (66.7)	3.251	0.071	0.535	2.889 (0.899, 9.283)
28-day mortality, n (%)	7 (31.8)	13 (48.2)	1.338	0.247	0.338	1.990 (0.616, 6.427)
Overall death, n (%)	11 (50.0)	20 (74.1)	3.023	0.082	0.512	2.857 (0.861, 9.483)
After controlled						
90-day mortality, n (%)	4.8 (21.8)	17.5 (64.6)	8.607	0.003	0.958	6.549 (1.850, 23.180)
In-hospital mortality, n (%)	3.9 (17.7)	14.9 (55.2)	6.944	0.008	0.846	5.730 (1.565, 20.976)
28-day mortality, n (%)	3.2 (14.4)	11.0 (40.7)	4.235	0.040	0.616	4.079 (1.036, 16.070)
Overall death, n (%)	5.2 (23.4)	18.5 (68.4)	10.456	0.001	1.012	7.085 (1.999, 25.105)

*OR* odds ratio, *SMD* standardized mean difference

## Discussion

*Pneumocystis jirovecii* pneumonia is a life-threatening complication following immunosuppressive therapy. When TMP/SMX treatment failure occurs, clinicians tend to blame it on the limited efficacy of TMP/SMX, and hastily change to a second-line drug without contemplating the real cause. Also, data on the efficacy of different second-line drugs remain limited. Therefore, the need to evaluate the risk factors for TMP/SMX treatment failure and consider alternative regimens has increased. We demonstrated that factors indicating co-infection were related to a poor response to first-line TMP/SMX treatment.

The factors related to first-line TMP/SMX treatment failure include symptom triad (breathlessness at rest, persistent fever and cough), treatment with invasive mechanical ventilation, coinfection with CMV and bacteremia. According to the guidelines for classifying the severity of PCP in HIV and non-HIV patients, the clinical features of severe PCP include breathlessness at rest, persistent fever and cough, PaO_2_ < 8 kPa in arterial blood gases, oxygen saturation < 91% at rest on air, and extensive interstitial shadowing [12]. We did not summarize the radiological findings, but the symptom triad and treatment with invasive mechanical ventilation indicated that the patients were in a severe condition [12]. Similarly, in Qing Yu’s study, CMV infection was proven to be associated with severe dyspnea and lower PaO_2_/FiO_2_, which also indicated severe PCP [13]. There are no published articles reporting the relationship between the severity of PCP and the treatment efficacy of TMP/SMX, but from our study, we may conjecture that severe PCP indicates a subgroup of patients who will have a poor response to first-line TMP/SMX therapy. In addition, both concurrent CMV infection and bacteremia were positively associated with mortality in non-HIV PCP patients, consistent with the results of published articles [13, 14]. Therefore, TMP/SMX treatment failure might be due to secondary infection rather than actual treatment failure.

To date, there are no high-quality guidelines or randomized controlled trials with a high grade of evidence supporting any second-line PCP treatment. The 6^th^ European Conference on Infections in Leukemia published guidelines for the PCP treatment of non-HIV-infected hematology patients in 2016, there was no second-line intervention with a grade A strength of recommendation. Primaquine plus clindamycin and pentamidine were the two alternatives having a grade B strength of recommendation [15]. According to Helweg-Larsen and colleagues, primaquine plus clindamycin had similar second-line therapy survival rates compared with TMP/SMX, while pentamidine was associated with a greater risk of death [16]. Similarly, Kim and colleagues demonstrated that the response rate of patients to C-P was higher than that of patients to pentamidine (64% vs. 11%, p = 0.03) [17]. Koga M et al*.* concluded that primaquine plus clindamycin appears to be a better candidate as a salvage treatment than pentamidine or atovaquone [18]. In our research found there was no parameter significantly associated with better second-line treatment outcome. Among clindamycin, primaquine and caspofungin, no drug or drug combination strategy was shown superior to others in terms of response and survive.

In recent years, caspofungin, a novel antifungal agent that inhibits the synthesis of β-1,3-glucan in the cell wall, has been proven effective in the treatment of PCP infection [19, 20]. Combined with TMP/SMX in the first-line setting, the positive response rate was significantly better and the all-cause in-hospital mortality rate was significantly lower compared with TMP/SMX monotherapy. The number of adverse events was not increased [21]. However, caspofungin acts on cyst forms of *P. jirovecii* only [19]. Furthermore, caspofungin was significantly less effective in second-line treatment than in first-line treatment in terms of both the mortality rate and response rate [22, 23]. Additionally, caspofungin combined with clindamycin was used for PCP treatment in recent studies [24, 25]. In our study, we found that the 90-day mortality and other secondary outcomes were higher in the caspofungin group. The cause of worse prognosis could be attributed to the greater severity and higher percentage of bacteremia in patients receiving caspofungin. Meanwhile, in recent years, it was demonstrated that caspofungin had better efficacy in PCP patients having higher BDG levels [11]. However, in our study, the BDG level was lower in the caspofungin group than in the noncaspofungin group, indicating a non-optimized patient population. Considering the poor accessibility, relatively high cost and intravenous route of administration of caspofungin, we suggest C-P conbination as a preferred choice for second-line non-HIV PCP treatment in mainland China if co-infection has been carefully ruled out. Although its efficacy is not significantly better in our study, its efficacy should be further tested in future investigation with larger sample size.

Our study has several limitations. First, it is a single-center retrospective study with a limited sample size and without a randomized controlled design; thus, the results should be extended to the whole patient population with caution. Prospective studies with larger sample sizes are warranted in the future. Second, because there are no widely accepted guidelines for second-line PCP treatment, the selection of drugs was complicated in our study. Third, the first-line TMP/SMX treatment success rate was lower than previous work [17], probably due to selection bias in our hospital, since PUMCH is the last choice for many critically ill patients in mainland China.

## Conclusion

CMV infection and bacteremia were risk factors significantly associated with treatment failure of TMP/SMX, the response and survival rates of second-line treatment, including clindamycin, primaquine, and caspofungin, were poor, maybe clindamycin plus primaquine as second line treatment was better than other treatment strategies. These results suggest that clinicians should carefully evaluate whether the treatment of TMP/SMX has failed due to a coinfection rather than hastily changing to a second-line drug when the patient worsens.

## Data Availability

The datasets used in the study are available from the corresponding author on reasonable request.

## Abbreviations

BDG: (1–3)-Beta-D-glucan
C: Clindamycin
C-P: Clindamycin plus primaquine
C-Ca: Clindamycin plus caspofungin
C-P-Ca: Clindamycin plus primaquine plus caspofungin
CI: Confidence interval
CMV: Cytomegalovirus
HIV: Human immunodificiency virus
IMV: Invasive mechanical ventilation
IS: Immunosuppressant
LY: Lymphocyte
LDH: Lactate dehydrogenase
OR: Odds ratio
PCP: Pneumocystis jirovecii pneumonia
p T/S: Preventive T/S to 2nd days
RID: Rheumatic immune disease
SD: Standard deviation
SMD: Standardized mean difference
SS: Sufficient steroid
TMP/SMX: Trimethoprim/sulfamethoxazole
TS: Typical symptoms of simultaneous breathlessness at rest, persistent fever and cough
T/S to 2nd days: TMP/SMX to second-line therapy days
WBC: White blood cell
